# Hearing difficulties, ear-related diagnoses and sickness absence or disability pension - a systematic literature review

**DOI:** 10.1186/1471-2458-12-772

**Published:** 2012-09-12

**Authors:** Emilie Friberg, Klas Gustafsson, Kristina Alexanderson

**Affiliations:** 1 Division of Insurance Medicine, Department of Clinical Neuroscience, Karolinska Institutet, Stockholm, SE-171 77, Sweden

**Keywords:** Sick leave, Disability pension, Hearing difficulties, Systematic literature review

## Abstract

**Background:**

Hearing difficulties is a large public health problem, prognosticated to be the ninth leading burden of disease in 2030, and may also involve large consequences for work capacity. However, research regarding sickness absence and disability pension in relation to hearing difficulties is scarce. The aim was to gain knowledge about hearing difficulties or other ear-related diagnoses and sickness absence and disability pension through conducting a systematic literature review of published studies.

**Methods:**

Studies presenting empirical data on hearing difficulties or ear-related diagnoses and sick leave or disability pension, published in scientific peer-reviewed journals, were included. Studies were sought for in three ways: in literature databases (Pub-Med, Embase, PsycInfo, SSCI, and Cochrane) through March 2011, through scrutinising lists of references, and through contacts. Identified publications were assessed for relevance and data was extracted from the studies deemed relevant.

**Results:**

A total of 18 studies were assessed as relevant and included in this review, regardless of scientific quality. Fourteen studies presented empirical data on hearing difficulties/ear diagnoses and sick leave and six on these conditions and disability pension. Only two studies presented rate ratios or odds ratios regarding associations between hearing difficulties and sick leave, and only two on hearing difficulties and risk of disability pension. Both measures of hearing difficulties and of sick leave varied considerable between the studies.

**Conclusions:**

Remarkably few studies on hearing difficulties in relation to sickness absence or disability pension were identified. The results presented in them cannot provide evidence for direction or magnitude of potential associations.

## Background

Hearing difficulties is a major public health concern [1-5], estimated to be the ninth leading burden of disease worldwide in the year 2030 [4]. Partial or complete hearing loss as well as other ear-related diagnoses such as vertigo or tinnitus can have consequences for work capacity. Sick leave or permanent marginalization from the labour market in terms of disability pension due to these diagnoses are, however, not much discussed or studied, although interventions regarding this are warranted and need to be based on scientific knowledge [6].

Known causes of hearing difficulties include various factors such as age, noise exposure, heredity, infections, health, and socio-economic factors [7,8]. Hearing difficulties as well as other ear-related diagnoses such as tinnitus, may lead to increased levels of stress, partly through the limited possibilities to be involved in ordinary conversions and the constant strain to hear [9]. This could potentially affect the risk of sickness absence and/or disability pension. Vertigo, balance related difficulties are also an important health problem that might lead to sick leave and exclusion from the labor market [10].

There are several indications of gender differences in the occurrence of hearing difficulties [9] as well as in sickness absence [11], why, gender stratified data regarding these aspects would be of interest.

Sickness absence has a large impact on society, employers, work sites, as well as on the individual and his or her family [6,12]. As most individuals with a disease or an injury can work, sickness absence is seldom a good measure of morbidity on a population level [6], however, it is a good measure of the social consequences of morbidity in terms of work incapacity [6,13]. In order to take adequate measures to prevent marginalisation among people with hearing difficulties, knowledge regarding the magnitude of such sickness absence and disability pension, is needed. Therefore, the aim of this study was to establish the current knowledgebase on hearing difficulties and other ear-related diagnoses and sickness absence and/or disability pension though conducting a systematic literature review of such studies.

## Methods

The systematic literature review was conducted in accordance to the PRISMA guidelines [14], in the following five steps; searches for studies, assessment of relevance of identified publications, data extraction, categorization of studies and synthesis of results from included studies. The following inclusion criteria were applied: Studies presenting empirical data on hearing difficulties or other ear-related diagnoses as well as on sick leave or disability pension published in scientific peer-reviewed journals. Both self-reported difficulties as well as ear-related diagnoses were included, to provide a wide inclusion and try to capture disorders and difficulties, which if diagnosed, would fall in the chapter of “Diseases of the ear and mastoid process” according to the “International Statistical Classification of Diseases and Related Health Problems, Tenth Revision” (ICD-10) [15].

Studies were sought for in the following three ways, no language or time restrictions were imposed:

1.) Searches, through March 2011, of the literature databases Pub-Med, Embase, PsycINFO, SSCI (Social Sciences Citation Index), and Cochrane using combinations of the following keywords: "sick leave" OR "sick-leave" OR "sickness absence" OR "sickness absent" OR "absenteeism" OR "return to work" OR "return-to-work" OR "disability pension" OR "work ability" OR "work inability" OR "work capacity" OR "work incapacity" OR "sickness benefit" OR "incapacity benefit" OR “work disability” OR “disability leave” OR “work disabled” AND "hearing" OR "deaf" OR "tinnitus" OR “vertigo” OR “auditory”. The combination of the sick-leave and hearing terms gave a total of 1229 hits (duplicates included) in PubMed (sick-leave terms and: hearing 607; deaf 160: tinnitus 46; vertigo 96; auditory 320), 2436 in Embase (sick-leave terms and: hearing 1012; deaf 443; tinnitus 101; vertigo 300; auditory 580), 51 in PsycINFO (including book chapters) (sick-leave terms and: hearing 10; deaf 8; tinnitus 0; vertigo 0; auditory 33), 1101 in SSCI (sick-leave terms and: hearing 163; deaf 119; tinnitus 38; vertigo 49; auditory 732), and 737 in Cochrane (sick-leave terms and: hearing 225; deaf 15; tinnitus 46; vertigo 66; auditory 385).

2.) Scrutinising lists of references of all studies deemed relevant. This gave two additional studies.

3.) Through contacts, i.e. asking other researchers if they knew about possible relevant studies. One additional study was identified.

The searches were conducted independently by two persons (E. Friberg and K. Gustafsson). All identified publications were assessed for relevance according to the above inclusion criteria. Titles and abstracts were read independently by at least two authors. In case of uncertainty or disagreement, the full article was read and, if needed discussed until consensus was reached. All relevant studies were included, irrespective of their scientific quality. Information regarding study design, population, measures, data collection, analyses performed, and relevant results (where applicable, the most adjusted result was extracted) were extracted from the included studies. Thereafter, the studies were categorized with regard to study design, type of study group (i.e. population, employees, or patients), and outcome. Finally, results from the included studies were compiled.

## Results

**Figure 1:**
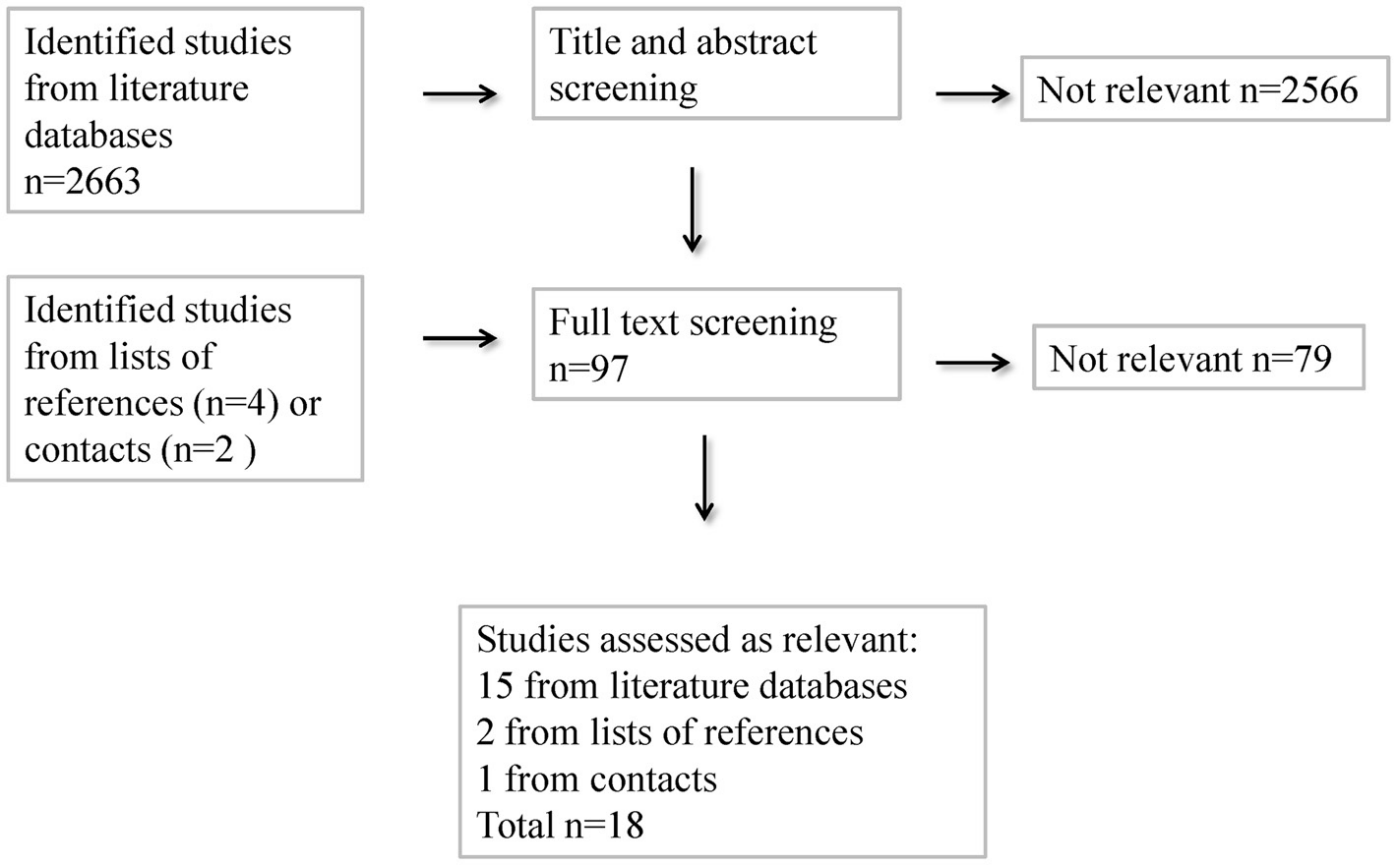
Number of identified publications and excluded, included, and relevant studies.

**Figure 2:**
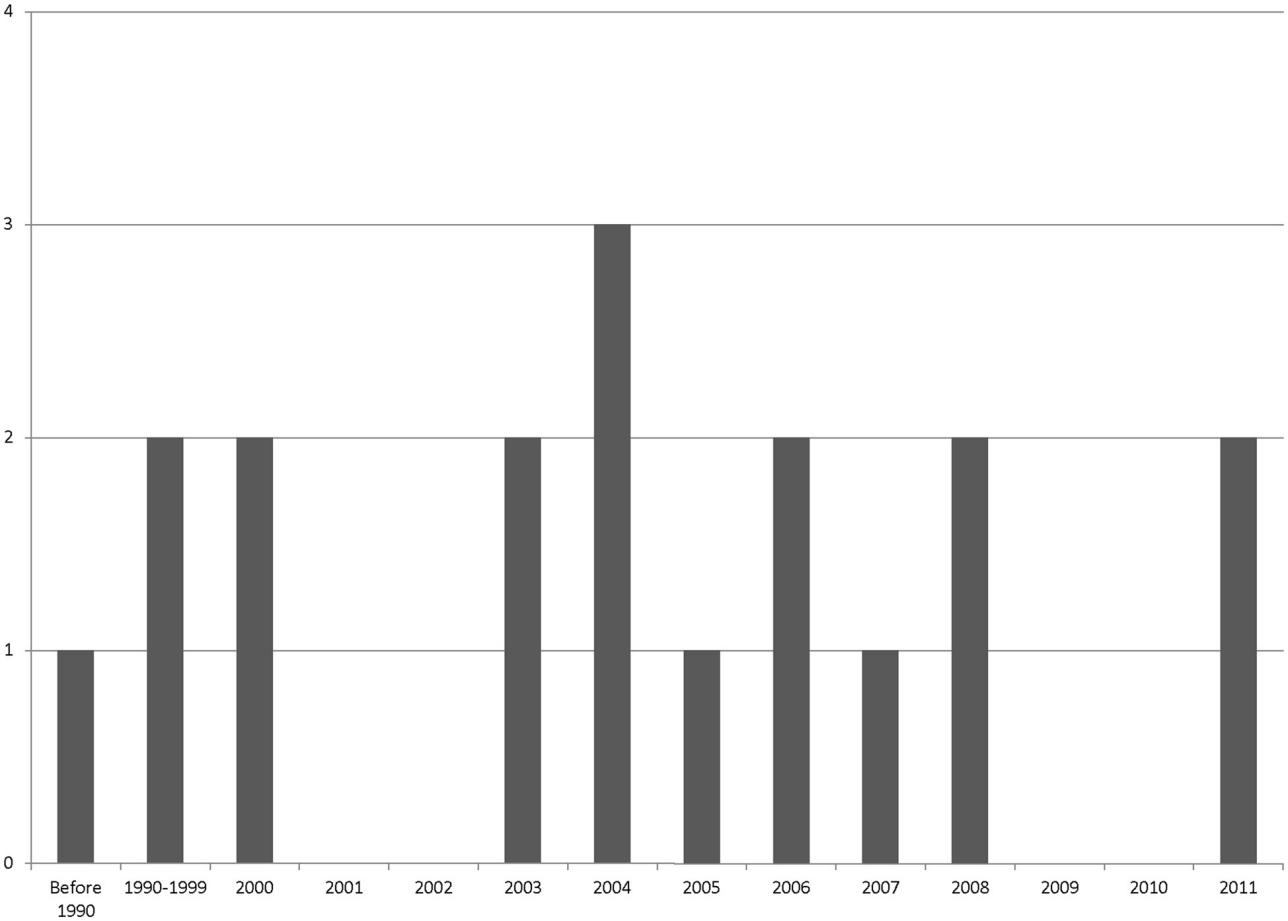
Number of studies published, by year.

When excluding double hits from different databases, a total of 2663 publications were identified, of which 18 were assessed as relevant (Figure 1), that is, they disclosed data on hearing difficulties or other ear-related diagnoses and on sick leave [10,16-28] and/or disability pension [17,18,29-32] (Table 1). The included studies were conducted in eight different countries (Sweden, USA, UK, Germany, Netherland, France, Norway and Poland, Table 1). All included studies were published in English and only three were published before the year 2000 (Figure 2). The majority (56%) of the studies had a cohort design, five (28%) had a cross-sectional design, and three were intervention studies (Table 2).

**Table 1:** Summaries of each of the 17 included studies, regarding included data on sickness absence, presented by type of study (cohort, intervention, cross sectional), in chronological order, by first author

**First author**	**Aim of the study**	**Study population.**	**Hearing diagnoses/ symptom**	**Type of data**	**Measures used of:**	**Results, regarding sickness absence**	**Results regarding disability pension**
**Year**		**Study participants (% of men (♂)).**		**Years of follow-up.**	**-Sick leave**		
**Country**		**Mean age (age range).**		**Years of data collection**	**-Disability pension**		
**Prospective cohort studies**							
Carlsson [16]	Investigate relation between sudden senorineural hearing loss and quality of life, psychosocial consequences and audiological rehabilitation.	588 (response rate 63%).	Sudden sensorineural hearing loss	Questionnaire data.	-Proportion of patients on sick leave and grade of sick leave.	10% upheld sick-leave benefits before the sudden sensorineural hearing loss, 29% directly after and 27% after ≥7 years, most of them for full time	
2011				7 years.			
				?			
Sweden		369 patients (56% ♂). 277 with information about sick leave.					
		Mean age 59 (13–91).					
Gustafsson [17]	Assess risk of being granted a disability pension among people with sick leave due to otoaudiological diagnoses compared to other sickness absentees.	231 499.	Otoaudiological diagnose as the reason for sick leave.	Data from sickness certificates and from registers.	- No. of individuals with a sick-leave spells (>7 days) due to otoaudiological diagnoses in 1985.	162 men with a new sick-leave spells due to otoaudiological diagnoses and 18 151 men with sick-leave spells due to other diagnoses.	RR for disability pension 1.42 (95% CI 1.23-1.64) among people on sick-leave with otoaudiological diagnoses compared to all other on sick leave, adjusted for age and sex.
2011		40 786 (45% ♂).					
Sweden		Age range 16–64.					
				11 years.			
				1985–96.			
						353 women with a new sick-leave spell due to otoaudiological diagnoses and 22 120 with sick-leave spells due to other diagnoses.	
					- No. and rate of people granted disability pension with different diagnoses.		
Skøien [18]	Assess incidence of vertigo in long-term sickness absence and to identify sociodemographic diagnostic predictors for transition into disability pension	1 939 355.	Vertigo (ICD10: H82) or dizziness (ICD10: N17) diagnoses.	Register data.	-No. of people on long-term sick leave (≥8 weeks) due to vertigo or dizziness diagnoses	282 women were on long-term sick leave due to vertigo, among 920 139 women eligible for sickness absence benefits and 134 men among 1 1019 216 eligible.	24% of men and 23% of women obtained a disability pension, Those with diagnose ICD10:H82 had a RR 1.5 (95% CI 1.1-1.9) for disability pension compared to those with diagnose ICD10:N17.
2008		1020 long-term sickness absentees, due to vertigo or dizziness (32% ♂).		5 years			
Norway							
				1997–2002.			
		Age range 16–62.					
					- No. granted disability pension		
Bjerlemo [19]	Follow the recovery process and explore the disease impact on sick-leave in patients with acute unilateral vestibular loss	All 44 patients from three hospitals, 27 responded (48% ♂).	Unilateral vestibular loss	Questionnaire data.	-Being on sick leave	At onset, 95% were on sick leave, after one month 26%; and after 6 months 22%.	
2006							
				6 months.			
Sweden							
				?			
		19 patients with information about sick leave.					
		Mean age 52 (16–70).					
Andersson [27]	Examine occupational status after 5 years, related to working hours, sick leave, pension, unemployment among patient with tinnitus.	189 tinnitus patients (77% response rate =146) (47% ♂).	Tinnitus	Medical files and questionnaire data.	-Being sickness absent at admission and at follow up (self reported).	No. of sickness absentees had decreased (13 vs. 6 subjects) at follow up.	
2000							
Sweden							
		142.					
		Mean age 56.4, age range 22–83.					
				Average 4.9 years (3–10 years).			
				1988–1995.			
Holgers [26]	Investigate risk factors for severe tinnitus measured as absence from work related to tinnitus of more than one month during the 18 month period	172 tinnitus patients, 127 (74%) completed questionnaire.	Tinnitus	Questionnaire and register data.	-Absent from work >1 month due to tinnitus.	18 patients had been absent from work >1 month during the study period.	
2000							
Sweden				18 months.			
		79 patients not on old age pensioned (?).		?			
		Mean age ♀ 75, ♂ 52.					
Starzynski [32]	Occupational diseases and consequences such as disability pension	86 871 cases of occupational diseases, 10 278 also with consequences.	Noise-induced hearing loss	Register data.	-No. granted disability pension among individuals with different occupational diseases		Among individuals with the occupational disease: noise induced hearing loss, 1990 (62%) had been granted disability pension.
1993				?			
Poland							
				1990–1992.			
		3228 noise induced hearing loss (?)					
		?.					
**Retrospective cohort studies**							
Hagberg [21]	To determine the incidence of tinnitus, hearing impairment and musculoskeletal disorders among musicians, and the relation to practicing hours and instrument type.	655 musicians (dropout due to: death 2%, emigration 6%, or non response 29%).	Tinnitus, hearing impairment	Questionnaire data.	-No. having been on sick-leave due to hearing problems (tinnitus and/or impaired hearing)	13 individuals had been on sick leave due to hearing problems.	
2005							
				Questions regarding present, previous year, 5, 10, and 15 years ago.			
Sweden							
		407 participants (54% ♂)					
				2000			
		Men: mean age 35 (23–49)					
		Women: mean age 34 (24–57).					
Sewell [30]	Examine the prevalence of hearing loss among Union Army veterans in the US by year, birth cohort and occupation and to compare civil war pension and contemporary disability programs by examining monthly dollar award.	Random sample of US Army veterans n = 35 747 (100% ♂).	Hearing loss from physical examination records	Medical records.	-Proportions of all pensions compensating for hearing loss.		Total 5891 or 33% of the individuals receiving a pension received compensation for hearing loss. Prevalence increased with time, age and later birth cohort.
2004							
				?			
USA							
				1862–1907.			
		17 722 receiving a pension.					
		?					
Rudin [28]	Long-term effects of otitis media on the general health, measured hearing loss and the wellbeing of the subjects.	A population-based sample of 945 men born in 1913 (81% response rate), 292 men born 1923 (75% response rate).	History of otitis media (self-reported).	Data from interviews and registers.	-No. of days and periods of sickness benefit.	Non-significant (p = 0.20) difference in number of days or periods of sickness benefits between history of otitis media compared to no history.	
1987							
Sweden							
				Register data for 1955–1973			
		927 (100% ♂).		1973.			
		Age 60 and 50 years-old.					
**Intervention studies**							
Gates [22]	Evaluate a portable low-intensity alternating pressure generator in controlling symptoms of Ménière’s disease.	62 Ménière’ patients, 30 in treatment group (33% ♂) and 32 in control group (31% ♂).	Ménière’s disease	“Diary”-data.	-No. of sick days (self-reported) during follow-up.	Median proportion of sick days all 4 months: treatment = 0.00, control = 0.02 (p = 0.02).	
2004.				4 months.			
USA.				2002–2003.			
				2002–2003.		Baseline mean proportion of sick days in the treatment = 0.05, and in the control = 0.07. At 4 months, 0.01 in both groups.	
		52 (84%) patients participated all 4 months.					
	A randomized, double-blinded, placebo controlled clinical trial.						
		Age range 33–71.					
Bjorne [24]	A cost-benefit analysis of reduction in treatment costs, sick leave and disability pension among working individuals with Ménière’s disease receiving treatment of tempemanibular and cervical spine disorders.	Ménière’s patients: 24 treated, 24 matched population-based controls. 4 (17%) dropped out.	Ménière’s disease	Register data.	-No. of sick-leave days due to Ménière’s or related symptoms 3 years before and 3 years after treatment.	Sick leave reduced from 1536 to 270 days, before and after treatment, compared to from 8 to 6 days for the control group.	
2003							
Sweden							
		19 patients and 19 controls (not on disability pension or old age pension) (47% ♂).					
		Mean age 52, age range 29–74.					
				3 years			
				1990–92.			
Joore [25]	To assess benefits in terms of gain in health-related quality of life and possible savings in terms of increased productivity and decreased use of medical services after hearing aid fitting in a population of moderately hearing impaired first-time hearing aid applicants.	80 hearing impaired patients.	Hearing loss.	Interview data.	-Absence from work due to health or due to hearing impairment	No reported absence from work due to hearing impairment or other health problems at baseline or at follow-up among those employed.	
2003				25 weeks			
Netherlands		10 with paid employment (100% ♂).		?			
		?					
**Cross-sectional studies**							
Neuhauser [10]	Assess burden of dizziness and quantify contribution of vestibular vertigo and non-vestibular dizziness	8318 random digit sample (response rate 52%) screened for history of dizziness; 1157 fulfilled criteria (had dizziness).	Vestibular vertigo.	Interview data.	-Reported sick leave among working individuals	Among individuals with vestibular vertigo, 40.6% reported sick leave compared to 14.7% among those with non-vestibular dizziness p < 0.001.	
2008							
						Higher rates among women.	
Germany				NA			
				2003.			
		1003 (87%) (?).					
		Age range 18–79.					
Ide [29]	Define level of hearing loss associated with ill-health retirement.	35 737 fire fighters.	Hearing loss in decibels	Questionnaire and medical data provided by work sites.	-No. and rate granted ill-health retirement due to hearing loss.		4% (n = 135 among 3366) of the ill health retirements were due to hearing loss.
2007		3366 whole time firefighters granted ill-health retirement (100% ♂).					
UK							
				NA			
		Mean age among firefighters granted ill health retirement due to hearing loss: 49 years, range 29–59.		1997–2002 (60 months).			
Kramer [20]	Compare the occupational performance of employees with hearing impairment and normal hearing.	Unknown	Post lingual hearing loss	Questionnaire data.	-No. of sick-leave days in the last 12 months and reason for sick leave.	Hearing impaired had increased risk of sick leave due to distress/strain OR 4.6 (1.3-16.5) compared to normal hearing, adjusted for age, sex, education, job demands/control/support, career satisfaction and type of contract.	
2006		150 hearing impaired employed (47% ♂), 60 normal hearing from the same workplaces (48% ♂).					
Netherlands				NA			
				2000–2001.			
		Mean age normal hearing 42.7, impaired hearing 45.3 (21–65).					
Chau [23]	Assess relationships of job, age and life conditions with causes and severity of occupational injuries.	880 construction workers (employed since at least 5 years) with non-fatal accident and sick-leave occasion and been seen by the physician.	Hearing impairment	Questionnaire data (filled in by the company physician).	-Sick leave >60 days, reported by occupational physician.	Hearing impaired individuals had a higher risk of sick leave (OR = 1.52). Analysis adjusted for age, BMI, sleep disorders, smoking, sporting activities, and occupation.	
2004							
France							
				NA			
				1995–96.			
		880 (100% ♂).					
		?					
Ide [31]	Count the causes of death and ill-health retirement, determine the emergence of any trends and examine the relationships between length of service and cause of ill health retirement or death among fire-fighters	505 ill-health retired fire fighters	Ear and mastoid diagnoses (ICD9:380–389) as primary diagnose.	Data from annual reports, management information system and medical records.	-No. with ill-health retirement		16 (3%) of the ill-health retirements were due to ear and mastoid diagnoses.
1998 UK							
				NA			
		488 (=96.6% with clinical notes) (100% ♂).		1985-1994			
		Mean age 43.75					

Abbreviation: *NA* Not Applicable.

**Table 2:** Categorization of included studies

**First author, year**	**Study design**			**Study group**			**Outcome**	
	**Cohort**	**Intervention**	**Cross-sectional**	**Population**	**Employed**	**Patients**	**Sick leave**	**Disability pension**
Carlsson [16], 2011	X					X	X	
Gustafsson [17], 2011	X			X			X	X
Neuhauser [10], 2008			X	X			X	
Skøien [18], 2008	X			X			X	X
Ide [29], 2007			X		X			X
Bjerlemo [19], 2006	X					X	X	
Kramer [20], 2006			X		X		X	
Hagberg [21], 2005	X				X*		X	
Gates [22], 2004		X				X	X	
Chau [23], 2004			X		X		X	
Sewell [30], 2004	X				X			X
Bjorne [24], 2003		X			X	X	X	
Joore [25], 2003		X				X	X	
Andersson [27], 2000	X					X	X	
Holgers [26], 2000	X					X	X	
Ide [31], 1998			X		X			X
Starzynski [32], 1993	X				X			X
Rudin [28], 1987	X			X			X	

* Former students.

There was considerable heterogeneity between the studies. Only four studies used population data, eight studies evaluated employed individuals, while six reported data on patients (Table 2). The hearing diagnoses/symptoms explored varied considerably between studies, including; sudden sensorineural hearing loss, otoaudiological diagnoses, unilateral vestibular loss, tinnitus, hearing impairment, Ménière’s disease, otitis media, vestibular vertigo, post-lingual hearing loss, vertigo, and ear and mastoid diagnoses. Data on hearing difficulties were in many of the studies self reported [10,16,19-21,28]. Outcome variables in the studies were different types of measures of sick leave and disability pension. Only four studies presented associations (i.e. rate ratios or odds ratios) between hearing difficulties and sick leave [20,23] or disability pension [17,18], respectively. Five studies presented data for patient groups [16,19,25-27] without any control or comparison group. Several studies were based on very small samples, for example, only six studies included more than 1000 individuals [10,17,18,29,30,32] and five studies included less than 100 individuals [19,22,24-26] (Table 1). The mean number of participants was 3926. Only two studies presented data stratified by sex [17,18] while six studies only included men [23,25,28-31]. A majority of the studies presented data for all-cause sick leave instead of diagnoses-specific sick leave whereas half of the studies on disability pension assessed diagnoses-specific disability pension (Table 3).

**Table 3:** Hearing difficulties in relation to sick leave and disability pension, categorization of included studies according to data reported

	**Data on sick leave**	**Data on disability pension**
Sick leave or disability pension due to hearing difficulties	Gustafsson [17], 2011	Ide [29], 2007
	Skøien [18], 2008	Sewell [30], 2004
	Hagberg [21], 2005	Ide [31], 1998
	Bjorne [24], 2003	
	Joore [25], 2003	
	Holgers [26], 2000	
Sick leave or disability pension, irrespective of diagnoses	Carlsson [16], 2011	Gustafsson [17], 2011
	Neuhauser [10], 2008	Skøien [18], 2008
	Bjerlemo [19], 2006	Starzynski [32], 1993
	Gates [22], 2004	
	Chau [23], 2004	
	Joore [25], 2003	
	Andersson [27], 2000	
	Rudin [28], 1987	

Note: the study by Kramer [20], 2006 reported sick leave due to distress/strain, i.e. not sick leave in general nor due to hearing difficulties.

### Sickness absence

In all, 45 850 individuals were included in the studies presenting data on sickness absence, 44 278 with known sex, of these 47% were men. The measures of sick leave were often self-reported, only in five studies were register data used [17,18,24,26,28].

Measures of sick leave varied considerably between studies, the following measures were used: proportion of patients on sick leave and grade (full or part time) of sick leave [16], sick leave >7 days due to otoaudiological diagnoses from national registers [17], number of individuals on long-term sick leave (>8 weeks) due to vertigo or dizziness [18], sick leave yes/no [19,27], sick leave due to hearing difficulties: yes, no, do not remember [21], absent from work more than one month due to tinnitus [26], number of days and periods of sickness benefit [28], number of days having to stay at home, leave work, or cancel a planned schedule, being bedridden or largely incapacitated during that day [22], number of absence days during a 3-year period before and after treatment from the national registers [24], absence from work due to health or due to hearing impairment [25], reported sick leave among persons with dizziness [10], self-reported number of absence days in the last 12 months and reason for sick leave [20], sick leave more than 60 days reported by physician [23].

Both studies [20,23] that presented data on associations between hearing difficulties and sick leave observed increased risks (OR 4.6; 95% confidence interval 1.3-16.5 and 1.52 (no confidence interval reported), respectively).

### Disability pension

In all, 66 610 individuals were included in the studies with information about disability pension, 63 382 with information on sex, of these 64% were men. The measure of disability pension varied somewhat, both all-cause disability pension [17,18,32] and diagnosis-specific disability pension [29-31] were evaluated. Disability pension was also called ill health retirement [29,31]. The following measures of disability pension were used: granted disability pension [17,18], granted ill health retirement due to hearing loss [29], proportions of pensions compensating for hearing loss [30], ill health retirement [31], and number of granted disability pensions among different occupational diseases [32].

The two studies presenting data on associations between hearing diagnoses as the reason for sick leave [17] or vertigo [18] and disability pension both observed an increased risk (RR 1.42; 95% CI 1.23-1.64 and RR 1.5; 95% CI 1.1-1.9, respectively).

## Discussion and conclusions

The aim of this literature review was to summarize research results from published studies that present empirical data on hearing difficulties or other ear-related diagnoses and sick leave or disability pension. The most striking finding from this literature review is the low number of published studies about sickness absence or disability pension due to hearing difficulties/diagnoses, considering how prevalent such hearing difficulties are. Secondly, the vast incongruity of the results, which partly can be explained by the large amount of different exposures, outcomes and measures examined in the different studies. We could only identify 18 relevant studies to include in this review, in spite of using several different search methods. Moreover, there are remarkable few relevant studies published on associations (i.e. rate ratios or odds ratios), between hearing difficulties and sick leave [20,23] or disability pension [17,18], however, all the four studies with such data, reported positive associations. Only two studies reported data stratified by gender, both showing a higher risk of disability pension among women with sick-leave due to audiological [17] or vertigo [18] diagnoses compared to men. We found no studies that assessed hearing difficulties as an outcome, after, for example, sick-leave or disability pension. The extremely large variation of data on hearing difficulties and on sickness absence and disability pension means that, at this stage, it is not constructive to compare results from the different studies regarding risks for sick leave or disability pension.

The strengths of this study are the systematic approach in searching and assessing studies, including a large number of keywords and the employing of a wide search strategy in well-known literature databases. Another strength is that two researchers independently conducted the searches. The very wide search made it possible to identify studies about hearing difficulties or other ear-related diagnoses, sick leave, and disability pension from different scientific disciplines. However, we cannot rule out that we might have missed studies; some scientific journals are not included in the literature databases; for example, newly established scientific journals. Moreover, several studies only reported the information without this being the actual aim of the study in question, which increase the risk that we might have missed some studies. Furthermore, the risk of publication bias cannot be ignored, small studies showing no associations or inverse associations might not have been published at all. Nevertheless, the number of retrieved publications with this wide search strategy resulted in very few relevant studies which indicate that sick leave and disability pension with hearing difficulties or other ear-related diagnoses have not been widely studied in scientific contexts.

In areas where more studies have been conducted, usually only studies that have a minimum level of scientific quality are included in systematic reviews. It became evident that the number of conducted studies in this field did not allow for excluding studies of lower scientific quality. Instead, the aim was to use all available studies to gain knowledge, why all studies identified were included, irrespective of quality.

In conclusion, there are remarkably few studies published on associations between hearing difficulties or other ear-related diagnoses and sick leave and/or disability pension and there are very large variations between the studies regarding design, study groups, analyses, and outcome measures. Nevertheless, all studies that disclosed results on associations between hearing difficulties or other ear-related diagnoses and sick leave or disability pension reported positive associations. However, at this stage, these results cannot be considered to provide enough evidence for such associations. This finding warrants further attention and not only are more, but also better studies needed before any conclusions can be made. There is an overall lack of prospective studies with long follow-up investigating the risk of diagnosis specific sick leave and disability pension among individuals with hearing difficulties or other ear-related diagnoses. Also intervention studies targeting the workplaces more directly would be of great interest.

## Competing interest

The authors declare that they have no conflict of interest.

## Authors’ contributions

Study concept and design: EF, KA. Systematic literature search: EF, KG. Interpretations of data: EF, KG, KA. Drafting of the manuscript: EF. Critical revision of the manuscript for important intellectual content: KG, KA. All authors have read and approved final version of manuscript.

## Pre-publication history

The pre-publication history for this paper can be accessed here:

http://www.biomedcentral.com/1471-2458/12/772/prepub
